# Preventive Practice and Associated Factors towards COVID-19 among College Students in Amhara Region, Ethiopia: A Cross-Sectional Study

**DOI:** 10.4314/ejhs.v31i1.2

**Published:** 2021-01

**Authors:** Abay Woday Tadesse, Negesse Melese Abebe, Sisay Eshete Tadesse, Mulugeta Chanie Wube, Ahmed Ali Abate

**Affiliations:** 1 Samara University, College of Medicine and Health Sciences, Afar Region, Samara, Ethiopia; 2 Dream Science and Technology College, Amhara Region, Dessie, Ethiopia; 3 College of Health Science, Debre Markos University, Debre Markos, Ethiopia Wollo University, College of Medicine and Health Sciences, Amhara Region, Ethiopia; 4 Wollo University, College of Business and Economics, Amhara Region, Ethiopia

**Keywords:** COVID-19, Knowledge, Attitude, Practice, Students, Amhara Region, Ethiopia

## Abstract

**Background:**

Ethiopia has taken unprecedented preventive measures like closure of higher education institutions to halt the spread of COVID-19. However, still, there is scarce information regarding the knowledge, attitude, and practice (KAP) of students towards COVID-19 pandemic. Thus, this study aimed to assess the KAP and associated factors of preventive measures against COVID-19 among students.

**Methods:**

A cross-sectional study was conducted on 422 students. The sample was proportionally allocated into the randomly selected four colleges, and the students were recruited using a systematic random sampling technique. Variables with p-value < 0.25 in the bivariate logistic regression analysis were entered into the multivariable logistic regression model.

**Results:**

This study involved 408 students with response rate of 96.6%. The levels of good knowledge, positive attitude and good practice towards COVID-19 were 69.6%, 56.6% and 65% respectively. After adjusting for covariates, being in the late adolescent age group (16–20), living with > 5 family size, and being single were predictors of knowledge level. Besides, being single, attending diploma (TVET) level trainings, and being year-two students were predictors of attitude levels. Similarly, urban residence, being regular students, and being year-one students were the independent predictors of practice level of students.

**Conclusion:**

In this study, only two-third of the students had good preventive practice level towards COVID-19, which is below the Organization's recommendation. Thus, the national, regional and local governments should develop effective and inclusive prevention strategies to address students who are at home due to COVID-19 pandemic.

## Introduction

Coronavirus Disease (COVID-19) is a viral disease caused by the beta coronavirus called severe acute respiratory syndrome coronavirus-2 (SARS-CoV-2) (1, 2). Corona Virus Disease-2019 (“COVID-19”) was first detected in December 2019 in Wuhan, China (3). Since COVID-19 has been declared a global pandemic by the World Health Organization (WHO), it has made a rapid spread across the world and it is causing high mortality and morbidity (1,4).

Globally, it causes an estimated number of 5.8 million cased and nearly half a million deaths at the end of May 2020 (5). In Ethiopia, according to the Ethiopian public health institute situational report, COVID-19 causes 5,846 cased and 103 death by the end of May, 2020 (6,7). Similarly, in Amhara Region, there were 307 cases and 5 death, which is the setting for this study.

Following this pandemic, nations across the globe have taken different preventive measures. These include movement restriction, confinement to home, and closure of schools and other social services (8–10). Hence, appropriate knowledge, attitude, and practices toward the preventive measures are mandatory to halt the spread of the COVID-19 outbreak in countries (11–14). However, previous studies revealed that communities have shown poor knowledge and negative attitude towards the preventive measures of COVID pandemic (15–17). On the other hand, most of the previous studies were predominately focused on the knowledge, attitude, and practices of health care workers toward the preventive measures of COVID-19 (18–20) that was not represent the KAP of college students.

Ethiopia has taken different prevention and control measures to halt the spread of COVID-19. These include school closure, stay at home, keep social and physical distances, putting hand washing basins in places where people use in common (banks, churches/mosques, markets), and establishment of state of emergency at the national level (21,22).

A study conducted in China revealed that adherence to prevention and control measures is an essential strategy to halt the spread of the outbreak, which is directly linked to the knowledge, attitudes, and practices (KAP) level of the population towards COVID-19 (23). In fact, the researchers were interested to assess the KAP of undergraduate students towards COVID-19. Besides, conducting survey studies may be helpful to generate rapid information regarding the KAP level of students towards COVID-19 who are at home due to the pandemic. Moreover, limited studies have been conducted to date to address the practice of students towards the prevention measures of COVID 19 in the country.

Therefore, this study was intended to assess the preventive practice and associated factors towards COVID-19 among college/university students.

## Methods and Materials

**Study setting and participants**: A community-based cross-sectional study was conducted from May 15–25, 2020 to assess knowledge, attitude, and practice level of students towards COVID-19. The study was conducted among students who were learning in the four randomly selected private and public colleges and universities namely; Dream Science and Technology College, Dandi Boru College, Unity University, and Dessie Health Science College. These higher institutions are found in South Wollo Zone, administration Dessie city. Dessie city administration is located 401Km away from the capital city of Ethiopia, Addis Ababa. The city has eight private colleges, one private university, and three public colleges which accommodate a total of 23,507 students in different fields of study.

All active students, registered in the second-semester academic calendar, and students of 16 and above years of age were included in this study. However, students who were seriously ill during the data collection period were not included in this study. The sample size was determined using a single population proportion formula with assumptions: 5% type I error, 95% Confidence intervals, 50% proportion for either knowledge, attitude, practice level since no study was one in Ethiopia prior to this study. Finally, the researchers added 10% to compensate for the non-response of participants and the final sample size became 422.

A simple lottery method was applied to select the higher education institutions. Proportional sample allocation was employed to get the required sample size from each selected university/colleges. And, then systematic random sampling technique was employed to get the study participants with their phone numbers from each teaching institution's registrar offices.

From the twelve colleges and universities found in Dessie city administration, three colleges and one university were randomly selected. The calculated sample size was proportionally allocated in each college based on the second-semester academic student number reports. To calculate the required number of participants from each college, we multiplied the total number of students actively learning in each college by the sampling fraction (n/N). The sampling fraction is approximately equal to six for all colleges. Accordingly, every 6^th^ participant was selected using systematic random sampling technique from each college registrar's office log-book.

Dependent variable were the preventive practice (good/poor) towards COVID-19 *while* independent variables were sociodemographic characteristics (age, residence, sex, marital status, religion, enrollment type, program, academic year, field of study, pocket income, family size), knowledge (good/poor), attitude (positive/negative), towards the preventive measures of COVID-19.

**Data collection tools and procedures**: The questionnaire was adapted from studies conducted before this study (24–26) and modified into context. The questionnaire was developed in English language. It has two main parts: sociodemographic and KAP related questions. The sociodemographic related variables consist of age, residence, sex, marital status, religion, enrollment type, program, academic year, field of study, average monthly pocket income, and family size. The second part of the survey assessed knowledge, attitude, and practice of students about COVID-19, which consists of nine items for knowledge, eleven items for attitude and eleven items for practice level (Supp. File 1). In the KAP questions with three options (Yes, No, and “I don't know”), correct responses were given one point while incorrect responses or “I do not know” responses were given zero points. Similarly, ‘moderate’ and ‘high’ were given one point while ‘very low’ and ‘low’ were given zero points.

The questionnaire was translated into the local language (Amharic) and back to English to keep its consistency. The data collection tool was pretested on 5% (21 participants) of the students who were learning in other than selected colleges (i.e. students from Yeju college located in Woldia town) and some amendments were made based on the pretest findings. The data was collected using both phone-call and personal interviews. Phone-call was used for students who are out of Dessie town. Trained health professionals who were working out of the selected colleges approached the study participants.

**Data management and analysis**: The data were cleaned, coded and entered into Epi data version 3.1 software and exported to SPSS version 24.0 for analysis. The descriptive analysis was done and the results were presented using texts, frequency tables, figures and median with Interquartile range.

Bivariate logistic regression analysis was done to assess the association between KAP and each independent variable. The socio-demographic factors with knowledge, attitude, and practice of preventive measures against COVID-19 were the included factors in the bivariate analysis. The independent variables with p-value less than 0.25 were considered in the final model. Correlation between independent variables was assessed but we did not find any correlation between independent variables. The model fitness was also checked using Hosmer-Lemeshow model fitness test. Finally, multivariable logistic regression analysis was done with backward elimination methods to control potential confounders and to identify the factors associated with the KAP of students towards COVID-19. A statistical significance level was declared at a P-value of less than 0.05.

The following operational definition are used in this study.

*Knowledge level*: students who correctly answered 70% or more of the knowledge questions were considered as students with good knowledge level while students who answered correctly below 70% of the knowledge questions were considered as having poor knowledge.

*Attitude level:* Students who correctly answered 70% or more of the attitude questions were considered as students with apositive attitude while students who correctly answered below 70% of the attitude questions were considered as students with a negative attitude.

*Practice level:* Students who correctly answered 70% or more of the practice questions were considered as students with good practice level while students who correctly answered below 70% of the practice questions were considered as students with poor practice.

## Results

**Characteristics of participants**: In this study, 408 participants were involved with a response rate of 96.6%. The median age of the participants was 21 years with three Interquartile Range (IQR). Of the total students, 155(38.0%) lived in the rural residence, 194(47.5%) were females, 215(52.7%) were learning TVET or diploma level training and 340(83.3%) were living with their families during the COVID-19 lockdown. In this study, the participants had a median of 5 total family size with 3 IQR (Table 1).

**Table 1:** Sociodemographic characters of college students in Amhara Region, Ethiopia, 2020

List of Predictors	Category of variables	Frequency (n=408)	Percentage (%)
**Age of participants** (in years)	16–20	166	40.7
	>20	242	59.3
**Residence**	Urban	253	62.0
	Rural	155	38.0
**Sex of the participants**	Male	214	52.5
	Female	194	47.5
**Marital status**	Single*	360	88.2
	Married	48	11.8
**Religion of the participants**	Orthodox	207	50.7
	Muslim	183	44.9
	Others+	18	4.4
**Type of Education enrollment**	TVET (Diploma)	215	52.7
	Degree (First)	193	47.3
**Program**	Regular	377	92.4
	Evening (Extension)	31	7.6
**Field of Study**	Health related	233	57.1
	Business related	129	31.6
	Technology related	46	11.3
**Academic year**	Year I	151	37.0
	Year II	180	44.1
	Year III	58	14.2
	Year IV+	19	4.7
**Living with;**	Families	340	83.3
	Relatives	28	6.9
	Alone	21	5.1
	Others++	19	4.7
**Total family size (including**	< 5	198	48.5
**extended families)**	5+	210	51.5
**Monthly income (in ETB)**	< 1000	349	85.5
	1000–1500	47	11.5
	> 1500	12	2.9

**Key:** Single* “currently not married”, Others+ (Protestant, Catholic), Others++ (friends, sister-in-law and son-in-laws)

**Figure 1:**
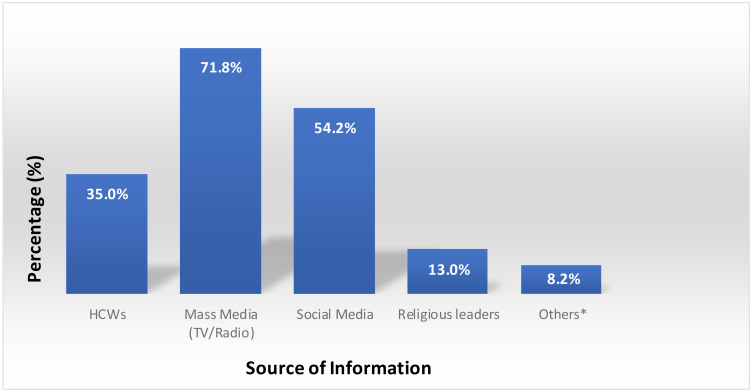
Source of information where college students acquired information regarding COVID-19 pandemic, June 2020, Ethiopia

**Source of information about COVID-19**: In this study, 293(71.8%) of the students had information about COVID-19 from mass media (TV, magazines, news paper, radio) and nearly fifty percent (54.2%) of the participants had information from social media (facebook, Instagram, whatsup and telegram) (Figure 1).

**Figure 2:**
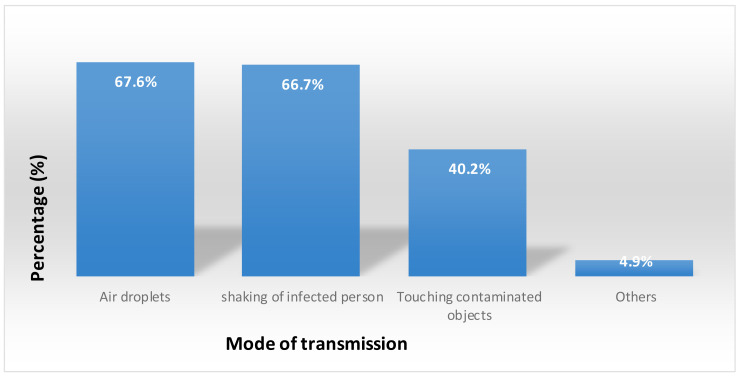
Students' knowledge on the mode of transmission of COVID-19, June 2020, Ethiopia

**Mode of Transmissions and sxymptoms of COVID-19**: In this study, 276(67.6%) of the students said that air droplets from the infected persons can transmit the infection of COVID-19 to healthy individuals. Similarly, 375(91.9%), 343(84.1%), and 324(79.4%) of the participants said that patients with COVID-19 can present with fever, dry cough, and shortness of breath respectively (Figure 2). In this study, 338(82.8%) of the students said that regular hand washing with water and soap can prevent COVID-19 pandemic. Similarly, 255(62.5%) of the participants said that we can deter the transmission of COVID-19 by covering of mouth and nose while coughing or sneezing.

**Students knowledge level and its associated factors**: In this study, 284(69.6%) [95% CI 65% 74.3%) of college students had good level of knowledge regarding COVID-19 pandemic. Selection of variables to be entered into multivariable logistic regression model was done based on clinical significance, predictor variables with p-value less than 0.25 in the bivariate logistic regression, and absence of multi-collinearity between independent variables. The selected covariates include age of respondents, residence, sex of participants, marital status, education enrollment type, study program, field of study, academic year, living conditions, and source of income for education were entered into the multivariable logistic regression analysis model. The multivariable logistic regression model was done with backward elimination methods.

In this study, the students who were in the age group (16–20 years) had twice higher odds of good knowledge level compared to those who were above 20 years old [AOR=1.78, 95% CI 1.07, 2.69]. Students who were living within more than five family members had 56% less likely to be knowledgeable about COVID-19 pandemic compared to those living within small family sizes [AOR=0.44: 95% CI 0.28, 0.68]. Moreover, students who were single had 2.3 times greater odds of good knowledge compared to married students [AOR=2.30: 95% CI 1.09, 5.55]. However, residence, sex of participants, education enrollment type, study program, field of study, academic year, living conditions, and source of income for education were not found significantly associated with knowledge level of students towards COVID-19 pandemic (Table 2).

**Table 2:** Factors associated with knowledge level of students, Amhara Region, Ethiopia, 2020

List of variable	Category of variables	Knowledge levels		COR (95% CI)	AOR (95% CI)

		Good (%)	Poor (%)		
Age category	16–20	126(44.4)	40(32.3)	1.67(1.07, 2.61)	1.78(1.07, 2.69)*
(in years)	> 20	158(55.6)	84(67.7)	1.00	1.00
Residence	Urban	182(64.1)	71(57.3)	1.33(0.86, 2.05)	1.24(0.77, 1.99)
	Rural	102(35.9)	53(42.7)	1.00	1.00
Sex of participants	Females	154(54.2)	60(48.4)	1.26(0.82, 1.93)	1.18(0.74, 1.89)
	Males	130(45.8)	64(51.6)	1.00	1.00
Marital status	Single	253(89.1)	107(86.3)	0.77(0.41, 1.45)	2.30(1.09, 5.55)*
	Married	31(10.9)	17(13.7)	1.00	1.00
Education	Diploma (TVET)	146(51.4)	69(55.6)	0.84(0.55, 1.29)	0.64(0.36, 1.12)
enrollment type	Degree (first)	138(48.6)	55(44.4)	1.00	1.00
Program	Regular	263(92.6)	114(91.9)	0.91(0.41, 1.99)	1.65(0.61, 4.48)
	Extension	21(7.4)	10(8.1)	1.00	1.00
Field of study	Health related	160(56.3)	73(58.9)	1.00	1.00
	Business	86(30.3)	43(34.7)	0.91(0.57, 1.44)	0.71(0.38, 1.32)
	Technology	38(13.4)	8(6.5)	2.17(0.96, 4.87)	2.60(0.93, 7.25)
Academic year	Year I	107(37.7)	44(35.5)	1.12(0.40, 3.14)	1.87(0.54, 6.46)
	Year II	123(43.3)	57(46.0)	0.99(0.36, 2.75)	2.05(0.58, 7.18)
	Year III	41(14.4)	17(13.7)	1.11(0.36, 3.41)	2.46(0.64, 9.43)
	Year IV+	13(4.6)	6(4.8)	1.00	1.00
Family size	< 5	121(42.6)	77(62.1)	0.45(0.29, 0.69)	0.44(0.28, 0.68)*
	5+	163(57.4)	47(37.9)	1.00	1.00
Living with;	Family	234(82.4)	106(85.5)	1.00	1.00
	Relatives	19(6.7)	9(7.3)	0.97(0.42, 2.18)	0.63(0.24,1,67)
	Alone	15(5.3)	6(4.8)	1.13(90.43, 3.00)	1.16(0.34, 3.910
	Others+	16(5.6)	3(2.4)	2.42(0.69, 8.47)	3.32(0.79, 13,95)
Monthly income for	< 1000	237(83.5)	112(90.3)	1.00	1.00
education (in ETB)	1000–1500	39(13.7)	8(6.5)	2.30(1.04, 5.09)	3.08(1.36, 6.95)*
	> 1500	8(2.8)	4(3.2)	0.94(0.28, 3.21)	1.27(0.36, 4.41)

**Key:** COR- Crude Odds Ratio, AOR- Adjusted Odds Ratio, * - P-value < 0.05, Others+: friends, sister-in-law and son-in-laws

**Students attitude towards COVID-19 and associated factors**: This study revealed that 230(56.4%) [95% CI 51.2%, 61%] of college students had positive attitude towards the prevention and control strategies of COVID-19 pandemic.

The multivariable logistic regression model was done with backward elimination methods. In this study, the odds of positive attitude among single students was 3-folds higher compared to married students [AOR=2.78, 95% CI 1.15, 6.68]. Students who were attending diploma (TVET) level trainings were 73% less likely to have positive attitudes towards COVID-19 prevention and control measures compared to those who were attending degree program trainings [AOR=0.27: 95% CI 0.17, 0.42]. Moreover, year-two students had 4-folds greater odds of positive attitude compared to year-four and above students [AOR=4.44: 95% CI 1.55, 12.68]. However, age of participants, residence, sex of participants, study program, field of study, living conditions, source of income for education, and knowledge level were not significantly associated with attitude level of students towards COVID-19 pandemic prevention measures (Table 3).

**Table 3:** Factors associated practice level of students in Amhara region, Ethiopia, 2020

List of variable	Category of variables	Practice levels		COR (95% CI)	AOR (95% CI)

		Good (%)	Poor (%)		
Age category	16–20	102(38.5)	64(44.8)	0.77(0.51, 1.16)	1.06(0.63, 1.76)
(in years)	> 20 years	163(61.5)	79(55.2)	1.00	1.00
Residence	Urban	189(71.3)	64(44.8)	3.07(2.01,4.68)	2.89(1.85, 4.53)*
	Rural	76(28.7)	79(55.2)	1.00	1.00
Sex of participants	Females	145(54.7)	69(48.3)	1.29(0.86, 1.94)	1.15(0.71, 1.85)
	Males	120(45.3)	74(51.7)	1.00	1.00
Marital status	Single	227(85.7)	133(93.0)	0.45(0.22, 0.93)	0.86(0.32, 2.27)
	Married	38(14.3)	10(7.0)	1.00	1.00
Education	Diploma (TVET)	131(49.4)	84(58.7)	0.68(0.45, 1.03)	0.81(0.45, 1.43)
enrollment type	Degree (first)	134(50.6)	59(41.3)	1.00	1.00
Program	Regular	238(89.8)	139(97.2)	0.25(0.18, 0.74)	0.26(0.18, 0.81)*
Extension	27(10.2)	4(2.8)	1.00	1.00
Field of study	Health related	146(55.1)	87(60.8)	1.00	1.00
	Business	85(32.1)	44(30.8)	1.15(0.73, 1.81)	0.71(0.38, 1,33)
	Technology	34(12.8)	12(8.4)	1.68(0.83, 3.43)	0.63(0.25, 1.53)
Academic year	Year I	85(32.1)	66(46.2)	0.15(0.12, 0.67)	0.17(0.14, 0.82)*
	Year II	113(42.6	67(46.9)	0.19(0.14, 0.88)	0.22(0.14, 1.05)
	Year III	50(18.9)	8(5.6)	0.73(0.14, 3.81)	0.84(0.15, 4.52)
	Year IV+	17(6.4)	2(1.4)	1.00	1.00
Family size < 5	126(47.5)	72(50.3)	0.89(0.59, 1.34)	0.72(0.45, 1.17)
	5+	139(52.5)	71(49.7)	1.00	1.00
Living with;	Family	207(78.1)	133(93.0)	1.00	1.00
	Relatives	24(9.1)	4(2.8)	3.85(1.31, 11.36)	3.50(1.13, 10.83)*
	Others+	34(12.8)	6(4.2)	3.64(0.89, 8.29)	1.42(0.44, 4.57)
Monthly income for	< 1000	219(82.6)	130(90.9)	1.00	1.00
education (in ETB)	1000–1500	38(14.3)	9(6.3)	2.51(1.17, 5.35)	1.21(0.44, 3.31)
	> 1500	8(3.0)	4(2.8)	1.18(0.35, 4.02)	0.44(0.19, 2.16)
Knowledge level	poor	78(29.4)	46(32.2)	0.88(0.57, 1.36)	1.04(0.62, 1.74)
	Good	187(70.6)	97(67.8)	1.00	1.00
Attitude towards	Negative	113 (42.6)	65(45.5)	0.89(0.59, 1.34)	0.89(0.54, 1.49)
	Positive	152(57.4	78(54.5)	1.00	1.00

**Key:** COR- Crude Odds Ratio, AOR- Adjusted Odds Ratio, * - P-value < 0.05, Others+ (friends, alone, sister/son-in-laws)

**Students practice level towards COVID-19 and associated factors**: This study revealed that 265(6%) [95% CI 60, 70.1%] of college students had good level of prevention practice regarding COVID-19 pandemic.

In this study, the students who were living in urban residence had 3-times greater odds of good practice level towards COVID-19 prevention and control measures compared to those who were living in rural residence during the pandemic [AOR=2.89, 95% CI 1.85, 4.53]. Regular program students were 74% less likely to have good practice on the prevention and control measures compared to extension (evening) program students [AOR=0.26: 95% CI 0.18, 0.81]. Finally, year-one students had 83% less likely good practice on the prevention and control measures compared to year-four students [AOR=0.17: 95% CI 0.14, 0.82]. However, residence, sex of participants, education enrollment type, field of study, living conditions, source of income for education, knowledge level and attitude towards COVID-19 prevention and control measures were not significantly associated with knowledge level of students towards COVID-19 pandemic (Table 4).

**Table 4:** Factors associated with attitude of students towards COVID-19

List of variable	Category of variables	Attitude levels		COR (95% CI)	AOR (95% CI)

		Positive (%)	Negative (%)		
Age category	16–20	87(37.8)	79(44.4)	0.76(0.51, 1.13)	0.62(0.37, 1.03)
(in years)	> 20 years	143(62.2)	99(55.6)	1.00	1.00
Residence	Urban	150(65.2)	103(57.9)	1.36(0.91, 2.04)	1.15(0.71, 1.85)
	Rural	80(34.8)	75(42.1)	1.00	1.00
Sex of participants	Females	124(53.9)	90(50.6)	1.14(0.77, 1.69)	1.37(0.85, 2.20)
	Males	106(46.1)	88(49.4)	1.00	1.00
Marital status	Single	208(90.4)	152(85.4)	1.62(0.88, 2.96)	2.78(1.15, 6.68)*
	Married	22(9.6)	26(14.6)	1.00	1.00
Education	Diploma (TVET)	93(40.4)	122(68.5)	0.31(.21, 0.47)	0.27(0.17, 0.42)*
enrollment type	Degree (first)	137(59.6)	56(31.5)	1.00	1.00
Program	Regular	214(93.0)	163(91.6)	1.23(0.59, 2.56)	1.26(0.48, 3.27)
	Extension	16(7.0)	15(8.4)	1.00	1.00
Field of study	Health related	110(47.8)	123(69.1)	1.00	1.00
	Business	91(39.6)	38(21.3)	2.67(1,69, 4.23)	1.58(0.85, 2.94)
	Technology	29(12.6)	17(9.6)	1.91(0.99, 3.66)	2.26(0.95, 5.41)
Academic year	Year I	73(31.7)	78(43.8	1.29(0.49, 3.38)	2.18(0.76, 63)
	Year II	118(51.3)	62(34.8)	2.62(1.01, 6.84)	4.44(1.55, 12.68)*
	Year III	31(13.5)	27(15.2)	1.58(0.55, 4.49)	1.77(0.57, 5.44)
	Year IV+	8(3.5)	11(6.2)	1.00	1.00
Family size	< 5	114(49.6)	84(47.2)	1.11(0.74, 1.62)	1.46(0.91, 2.34)
	5+	116(50.4)	94(52.8)	1.00	1.00
Living with;	Family	194(84.3)	146(82.0)	1.00	1.00
	Relatives	14(6.1)	14(7.9)	0.75(0.35, 1.63)	0.47(0.18, 1.22)
	Alone	11(4.8)	10(5.6)	0.83(0.34, 2.01)	0.67(0.22, 2.04)
	Others+	11(4.8)	8(4.5)	1.03(0.41, 2.64)	0.84(0.27, 2.66)
Monthly income for	< 1000	195(84.8)	154(86.5)	1.00	1.00
education (in ETB)	1000–1500	30(13.0)	17(9.6)	1.39(0.74, 2.62)	2.22(0.93, 5.26)
	> 1500	5(2.2)	7(3.9)	0.56(0.18, 1.81)	0.78(0.19, 3.14)
Knowledge	Poor	47(20.4)	77 43.3)	0.34(0.22, 0.52)	0.31(0.19, 0.48)*
	Good	183(79.6)	101 (56.7)	1.00	1.00

**Key:** COR- Crude Odds Ratio, AOR- Adjusted Odds Ratio, * - P-value < 0.05, Others+: friends, sister-in-law and son-in-laws.

The study found that the overall level of knowledge regarding COVID-19 pandemic among college students was 69.6% [95% CI 65% 74.3%). This finding is lower than studies conducted among Indian medical students (94.5%) (27), eight countries of five continents (80.8%) determinants (28), Malaysia (80.5%) (29), Sudan (90.6%) (30), and Pakistan (71.5%) (20). The discrepancy might be due to differences in cut-values used to categorize the knowledge levels, sample size, and sociocultural variables between study settings. However, this result is higher than a study conducted in Syrian residents (60%) (31), USA (58%) (32), Bangladesh (48.3%) (57.6%) (10%) (17, 33, 34), three Middle East countries (66.1%) (35), Makerere University Teaching Hospital (66%) (18), and Pakistan (51.8%) (36). The differences in level of knowledge have been subjected to variation in the cut-values (i.e. most of the previous studies used more than 80% to say good knowledge) while this study used 70% to categorized study participants with good level of knowledge. In addition, the discrepancies might be due to differences in sample size and study settings.

This study revealed that students in the late adolescent age group (i.e. 16–20 years) were twice more knowledgeable regarding COVID-19 compared to those who were above 20 years old. This finding is similar to a study done in Kingdom of Saudi Arabia (37), China (25), Medical college students in Uttarakhand, and India (24). Adolescents are very eager to know emerging new events including the new novel virus (COVID-19) than adults (38). Hence, students in the late age group are more knowledgeable compared to adults.

This study showed that students living with less than five family members had 56% less likely to have good knowledge of COVID-19 pandemic than to those living within small family sizes. This finding is consistent with a study conducted in Tanzania (39). This could be justified by the fact that students from small family size may spend most of their time by watching movies than families with large members who are obligated to have common source of information that will help the whole family members. Thus, these people are more likely to get the information disseminated by the government compared to their counterparts.

The finding of this study indicated that single students had 2.3 times greater odds of good knowledge compared to married students. This finding is supported by a study conducted in eight countries to assess knowledge level and its sociodemographic determinants (28). Hence, single students may have sufficient time (23) to acquire adequate information regarding COVID-19 compared to married individuals who may be busy in the care of the families.

This study revealed that 56.4% [95% CI 51.2%, 61%] of study participants had a positive attitude towards the prevention and control measures of COVID-19 pandemic. This finding is lower than studies conducted among Indian medical students (93.7%) (27), Syrian residents (63.5%) (31), Malaysian residents (83.1%) (29), Bangladesh (62.3%) (17), Sudan (81.8%) (30), 10 universities in Shaanxi Province, China (73.8%) (40), Uganda (72.4%) (41), and India (97.3%). However, this result is higher than the studies conducted in Pakistan (44%) (20), and Makerere University Teaching Hospital (21%) (18). The discrepancy may be subjected to variation in the cut-values to measure the positive and negative attitude levels. Besides, the discrepancies might be due to differences in sample size and study settings.

In this study, the odds of positive attitude among single students was 3-folds higher compared to married students. This finding is consistent with a study conducted in eight countries of five continents (28). Consequently, single students might have adequate time (23) to listen and search relevant information regarding COVID 19. Thus, single students could have positive attitude towards COVID-19 compared to married individuals who have busy time taking care of the families.

This study revealed that students who were attending diploma (TVET) level training were 73% less likely to have positive attitudes towards COVID-19 preventive measures compared to those who were attending degree program training. Moreover, year-two students had 4-folds greater odds of positive attitude compared to year-four and above students. When the education level of students increases, their attitude towards the preventive measures will increase. This is supported by the study conducted among Indonesian undergraduate students (42). Thus, students with degree level training could have a higher attitude towards the preventive measures of COVID-19 than students attending lower level training.

This study revealed that 65% [95% CI 60, 70.1%] of college students had a good level practice towards the prevention measures of COVID-19 pandemic. This finding is lower than a study done in Syria (73.8%) (31), Pakistan (80.5%) (36), Sudan (89.9%) (30), students in 10 universities in Shaanxi Province, China (87.9%) (40), and Uganda (85.3%) (41). However, this finding is higher than studies conducted in Bangladesh (55.1%) (17), and Pakistan (57.3%) (20). The differences in the practice of preventive measures could have been subjected to variation in the cut-off values to classify good or poor practice. For instance, most of the previous studies used above 80% scores to determine adequate practice while the current study used 70% and above to categorize study participants to good level of practice. In addition, the discrepancies might be due to differences in sample size and study settings.

This study indicated that students who were living in urban residence had 3-times greater odds of good practice level towards COVID-19 prevention and control measures compared to those who were living in rural residence during the pandemic. This finding is similar to studies conducted among Indonesian undergraduate students (42), Sudan (30), and Nepalese residents.

Moreover, this study revealed that regular program students were 74% less likely to have a good practice on the prevention and control measures compared to extension (evening) program students. Similarly, year-one students had 83% less likely good practice on the prevention and control measures compared to year-four students. This finding is similar to studies conducted among Indonesian undergraduate students (42). Therefore, when the year of study increases the level of practice also increases. Hence, year four students have a greater practice of preventive measures of COVID-19 compared to year-one students.

This study was done using a phone-call interview which may be prone to social desirability bias. Besides, the study did not involved adolescents in high schools and pre-college schools. Thus, it may not represent all of the adolescents in Dessie town. Moreover, the study also shares the limitations of a cross-sectional study design.

In conclusion, the overall students' KAP level towards COVID-19 was not comparable to the WHO recommended KAP scores for the general population, which is true of students.

After adjusting for covariates: being in the late adolescent age group, living with > 5 family size, and being single were predictors of knowledge level.

This study revealed that being single, taking diploma (TVET) level trainings, and being year-two students were predictors of attitude levels. Finally, residence, being regular students, and academic year were the independent predictors of the practice level of students. Therefore, the authors have recommended that the Ministry of Science and Higher Education [MOSHE], regional education bureau, and local governments have to develop effective strategies and interventions to address the identified gaps of KAP and its associated factors among students that will have direct negative impact on the prevention and control activities to halt the spread of the outbreak. Besides, this finding will help the private and public college administration to reach their students since the summary of the results were disseminated to all included colleges and other stakeholders.
